# A pilot study of digital bedside cards in the emergency room of a medical centre in Taiwan

**DOI:** 10.1038/s41598-023-34234-4

**Published:** 2023-04-28

**Authors:** Wei-Hsiang Liao, Shih-Yi Lee, En-Chih Liao, Wen-Han Chang, Tse-Hao Chen, Ming‑Kun Huang, Jui-Ping Lin, Weide Tsai, Fang-Ju Sun, Ding-Kuo Chien

**Affiliations:** 1 Department of Medicine, MacKay Medical College, New Taipei City, Taiwan; 2 Department of Emergency Medicine, MacKay Memorial Hospital, Taipei, Taiwan; 3 MacKay Junior College of Medicine, Nursing, and Management, New Taipei City, Taiwan; 4 Graduate Institute of Injury Prevention and Control, Taipei Medical University, Taipei, Taiwan; 5 Institute of Mechatronic Engineering, National Taipei University of Technology, Taipei, Taiwan; 6 School of Medicine, Taipei Medical University, Taipei, Taiwan; 7 Department of Medical Research, MacKay Memorial Hospital, Taipei, Taiwan

**Keywords:** Software, Health care, Engineering

## Abstract

The emergency room (ER) digital bedside card is a simple and important invention. It can be directly connected to the hospital information system to display important patient information in real time, reduce the workload of ER staff, improve their satisfaction, and provide useful information for patients and their families. We conducted a prospective study of ER staff using questionnaires and conducted Wilcoxon signed-rank test to compare before and after ER digital bedside card implementation in the Tamsui MacKay Memorial Hospital. Sixty participants of the ER staff joined the study before and after digital card implementation. After the ER digital bedside card was set up, the number of round trips from the nursing station to the ER bedside and the number of common questions asked by patients and their family members were significantly reduced. The cards reduced the response time for frequently asked questions by patients and their family members and significantly improved the satisfaction of ER staff. Our study showed that ER digital bedside cards reduced the workload of ER staff, provided patients and their families with useful information, and greatly improved ER staff satisfaction. This marks an important milestone in the future development of smart ER.

## Introduction

Emergency room (ER) crowding is a major problem in Taiwan because patients prefer to visit the ERs of medical centres even for simple medical problems^1^. The flow of ER patients is very high, and the beds change rapidly. After the ER physician visits a patient, the nurse assigns the patient to a bed in the observation area if necessary. During patients’ stay in the ER, they may need to go to the laboratory to have blood drawn, radiology for X-rays, and the physiological examination department to undergo gastroscopy or ultrasonography. Therefore, it is necessary to repeatedly transfer the patient out of the observation area, and when bringing the patient back, it is necessary to confirm that the patient is in the correct bed.

**Figure 1:**
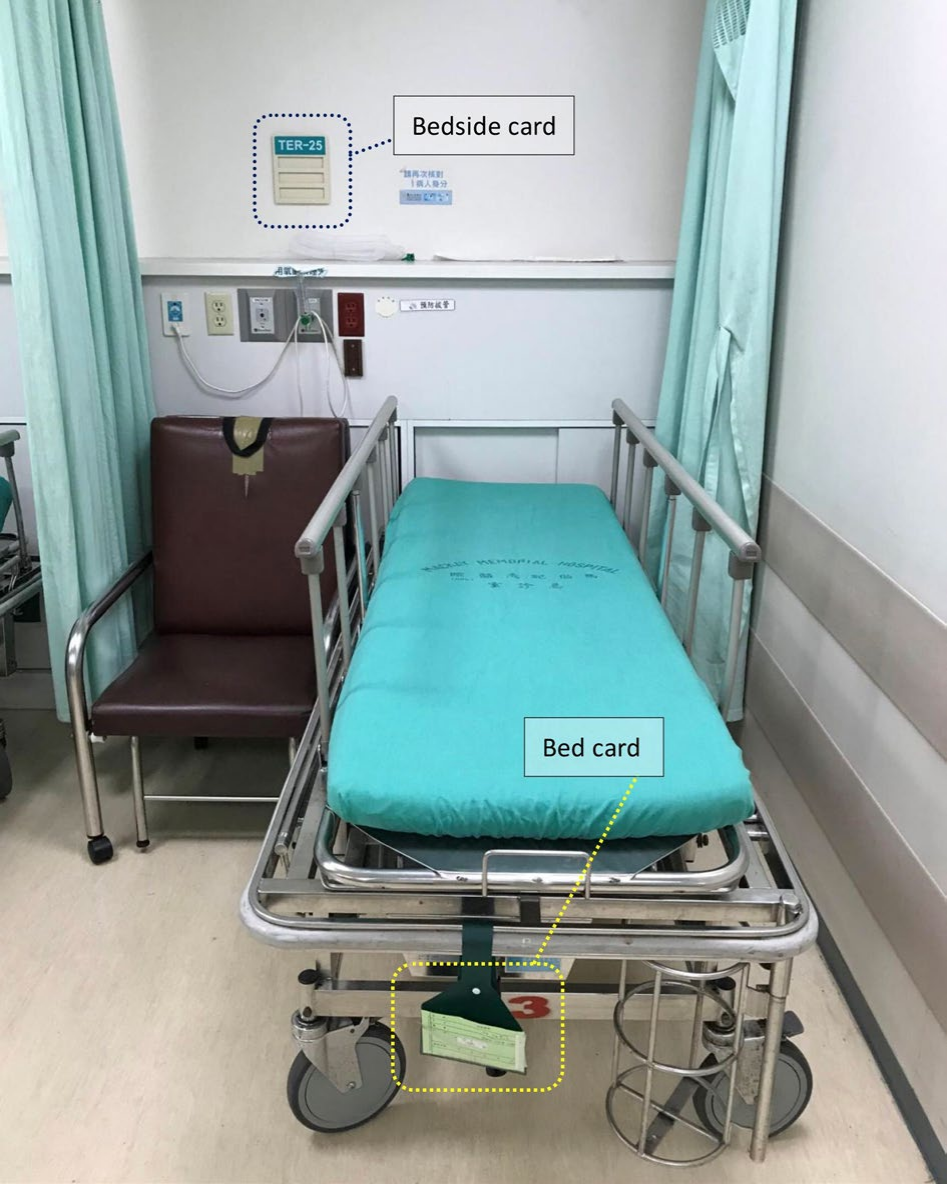
Traditional bedside card (blue dot) and bed card (yellow dot) in the emergency department.

In the past, the traditional ER bedside card was only a simple bed number on the wall above the headboard (Fig. 1). There was no ER digital bedside card that could provide real-time patient information and allow ER staff to confirm that the patient was in the correct bed^2^. Therefore, the nurse at the nursing station needed to tell the escort to which bed to return the patient. The escort returned the patient to the bed in the observation area based on memory and could not confirm whether the bed was correct.

**Figure 2:**
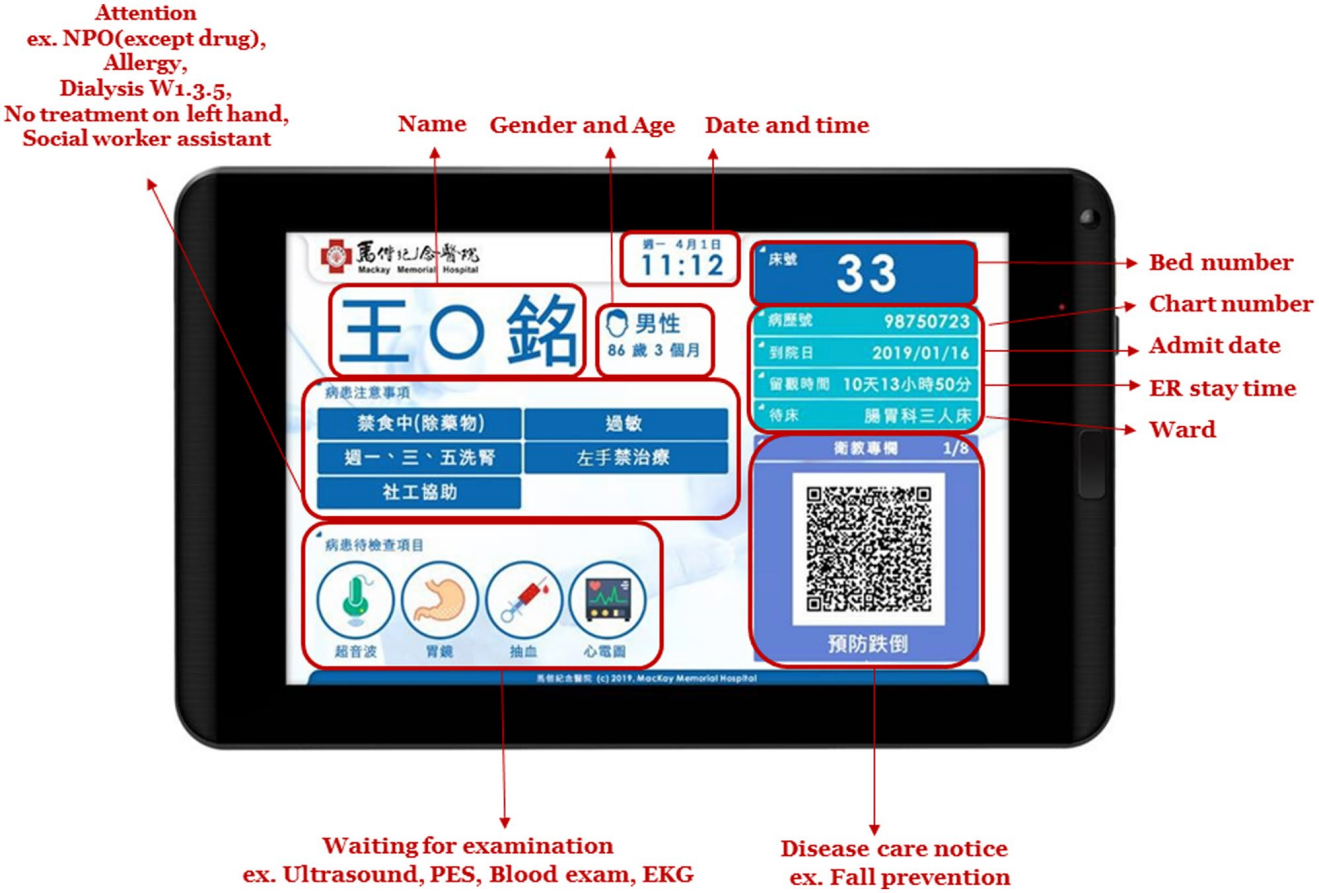
Contents of an ER digital bedside card.

**Figure 3:**
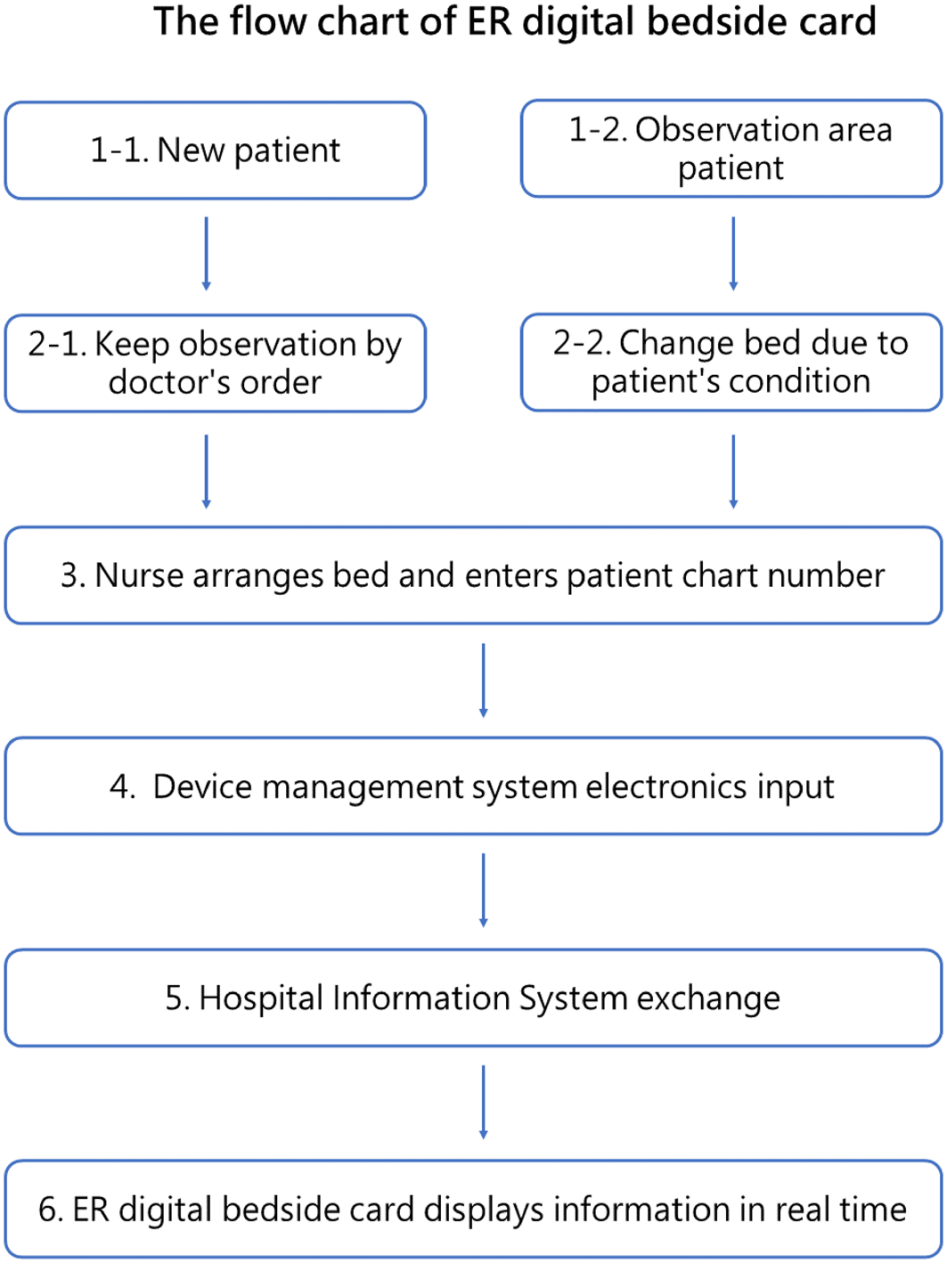
Flow chart of the ER digital bedside card.

Through MacKay Memorial Hospital's smart ER outreach programme, we set up an ER digital bedside card (Figs. 2 and 3) in September 2020, an electronic display device connected to the hospital information system (HIS) that can display real-time patient information and enhance patient identification by providing instructions such as nothing by mouth, do not resuscitate, the anticipated procedure (such as ultrasound, endoscope, blood exam, CT scan, or X-ray), and the admission status of the patient^2^. Patients and their families can quickly understand the patient’s current condition by viewing the digital bedside card. It can also help ER staff identify patients at their bedside and assist in administering the correct treatments to the correct patients.

In addition to helping patients be placed in the correct bed, the digital card can alert ER staff to important patient considerations. Furthermore, it can provide patients and their families with the patient’s current status, such as whether the patient can eat, which type of exam the patient is awaiting, whether hospitalization is needed, and whether patients are willing to be resuscitated if their condition worsens. There is also health education information available via QR codes. Finally, in addition to effectively identifying the bed’s location, the card can reduce the burden of online ER staff.

Based on a current literature search on 2023/02/05 using the term ‘digital bedside card’ on PubMed, 2 studies were found, but they were not connected to our concept. Using the term ‘bedside card’ in a search of 2000–2023 on PubMed, 96 studies were found, and two studies with the same title, ‘Design and Implementation of Multifunctional Interactive Electronic Bedside Card System for Inpatients Based on Internet of Things Technology’, appeared to include similar concepts. However, this paper was written in Chinese, it was set in the ward rather than in the ER^3,4^. Using the terms (bedside card) AND (emergency department) on PubMed, 9 studies were found, but there was no connection to our concept. There was a conference paper titled "An Electronic Hospital Bedside-Card Prototype Using RFID and DVB-MHP Platform", which appeared to be a similar concept but was also set in an inpatient room rather than an emergency room^5^.

The only literature related to digital bedside cards for the ER that has been published is our previous work^2^. We conducted this study in the hope that establishing an ER digital bedside card can reduce labour for ER staff, including the number of round trips from the nursing station to the ER bedside, the number of frequently asked questions by patients and their families, and the response time for answering questions and can improve ER staff satisfaction.

## Methods

At the beginning of 2018, the Department of Emergency Medicine of MacKay Memorial Hospital (MMH) cooperated with the digital development plan of MMH and initially conceived of setting up ER digital bedside cards. At the same time, we conducted this study, which was approved by the institutional review board (18MMHIS161e) of MacKay Memorial Hospital, Taipei, Taiwan. The idea was supported by the hospital, and a utility model patent (M585421) was obtained in Taiwan in October 2019. All methods were conducted in accordance with relevant guidelines and regulations (18MMHIS161e). Informed consent was obtained from all subjects.

### Study participants

The study participants included emergency physicians, nurses, nurse practitioners, and escorts.

There were 73 ER colleagues aged 21–63 years who participated in the pretest questionnaire in December 2019. The ER digital bedside card was set up completely in September 2020, and 83 ER colleagues participated in the posttest questionnaire in January 2021. A total of 60 ER colleagues participated in both the pretest and posttest questionnaires.

### Definition and data collection

The Supplementry material included a pretest and a posttest. The pretest was the questionnaire collected before the ER digital bedside card was established, whereas the posttest was the questionnaire collected after the ER digital bedside card was established and ER staff were familiar with the device.

The contents of the questionnaire included the following: job title; seniority; shift period; the number of patients cared for; whether the patient was in the wrong bed and the possible reasons; the number of round trips from the nursing station to the bedside; the number of questions from patients and their family members and what the questions were; the response time, defined as the time from the end of patient or family member asking a question to the end of an ER staff member’s answer to the question; and a statistical analysis of ER colleagues' satisfaction with traditional bedside cards and ER digital bedside cards.

With regard to the definition of the number of round trips from the nursing station to the bedside, going to the observation area to take care of one patient counted as one time, while going to the observation area to take care of three patients in a row counted as three times.

For the definition of the number of questions asked by patients and families, if a patient in the observation area asked one medical-related question, it counted as one time; if the patient asked three medical-related questions at a time, it counted as three times.

The response time was defined as the time from the end of a question to the end of an ER staff member’s answer to the question. For example, a patient said, “Can I eat something now?”, and a staff member found the answer from the computer at the station or the digital bedside card and replied, “Yes, you can eat now” or “No, you can’t”. The pretest response time was the time needed to return to the nursing station from the bedside to obtain information from the HIS and then return to the bedside to answer the patient's question. The posttest response time was the time needed to provide answers to the patient’s questions directly from the ER digital bedside card.

The questionnaire was distributed to colleagues by an ER researcher and nursing team leaders. If the participants had questions about the content, they could ask the researcher anytime during a weekday. The questionnaire stated, “Some questions in this questionnaire need to be answered based on your actual daily work situation. It is recommended to read the questionnaire questions first and then pay attention to the work situation on the next working day to facilitate completion. Thank you!”.

### Statistical analysis

Statistical Package for the Social Sciences (SPSS) Statistics 25.0 for Windows (SPSS Inc., Chicago, IL) was used for data management and statistical analysis. Wilcoxon signed-rank test examined the pretest and posttest differences. A *P* value < 0.05 was the criterion for statistical significance.

According to the central limit theorem (CLT)^6^, if a population has a mean μ and standard deviation σ and sufficiently large random samples are taken from the population with replacement, then the distribution of the sample means will be approximately normally distributed. In our study, the sample size was more than 30, and the estimates of analysis were robust. Therefore, we initially performed t-tests on two groups of continuous variables. However, we tested for normality of distribution using the Kolmogorov-Smirnov test or the Shapiro-Wilk test, and our data failed the normality test. Therefore, we use Wilcoxon signed-rank test instead of Paired t-tests to examine pretest and posttest differences.

In many trials, it is not uncommon to face multiple testing problems that can impact both type I and type II error rates, leading to an inappropriate interpretation of the trial results. However, most of the questions in our questionnaire involved different concepts. In addition, the repeated measurements were before and after tests, so there was little multiplicity in our study.

### Ethical approval

The authors are accountable for all aspects of the work in ensuring that questions related to the accuracy or integrity of any part of the work are appropriately investigated and resolved.

## Results

A total of 60 ER colleagues participated in both the pretest and posttest questionnaires. Wilcoxon signed-rank test was used to examine the pretest and posttest differences.

Table 1 shows a total of 60 ER colleagues who participated in both the pretest and posttest, including 4 ER physicians, 35 nurses, 6 nurse practitioners, and 15 escorts. The participants included 4 males and 56 females. The majority had seniority of 0–5 years, followed by > 16 years. Furthermore, the majority of the participants worked day shifts, followed by small night shifts.

**Table 1:** Demographic comparison before and after setting up the ER digital bedside card (N = 60).

	Pretest		Posttest	
	N = 60	%	N = 60	%
*Job title*				
Physician	4	6.7	4	6.7
Nurse	35	58.3	35	58.3
Nurse practitioner	6	10.0	6	10.0
Escort	15	25.0	15	25.0
*Gender*				
Male	4	6.7	4	6.7
Female	56	93.3	56	93.3
*Seniority*				
0–5 years	30	50.0	25	41.7
6–10 years	11	18.3	12	20.0
11–15 years	5	8.3	8	13.3
≧16 years	14	23.3	15	25.0
*Work hours*				
07:00–19:00	5	8.3	5	8.3
19:00–07:00	1	1.7	0	0
08:00–16:00	24	40.0	22	36.7
16:00–24:00	15	25.0	22	36.7
24:00–08:00	15	25.0	11	18.3

Table 2 shows that the number of round trips from the nursing station to the bedside was significantly reduced (44.05 ± 15.47 vs. 35.42 ± 11.66, *P* < 0.001) after setting up the ER digital bedside card.

**Table 2:** Comparison of the number of round trips from the nursing station to the bedside before and after setting up the ER digital bedside card.

	Pretest		Posttest		Pretest–posttest		Z	*P*
	M	SD	M	SD	M	SD		
The number of round trips	44.05	15.47	35.42	11.66	8.63	10.29	− 5.04	< 0.001

Table 3 shows that the total number of questions asked by patients and their family members was significantly reduced (56.73 ± 26.58 vs. 23.80 ± 10.19, *P* < 0.001) after setting up the ER digital bedside card. For further analysis, the questions that were frequently asked, such as “Can I eat”, “Wait for what”, “Wait for what kind of exam”, “Whether to be hospitalized”, “Current status”, and “Other”, were significantly reduced.

**Table 3:** Comparison of the number of questions from patients and their family members before and after setting up the ER digital bedside card.

	Pretest		Posttest		Pretest–posttest		Z	*P*
	M	SD	M	SD	M	SD		
Total number of questions asked	56.73	26.58	23.80	10.19	32.93	22.71	− 6.71	< 0.001
→ questions in detail								
Can I eat	11.78	5.78	4.30	2.28	7.48	5.27	− 6.63	< 0.001
Wait for what	13.70	7.80	5.70	3.13	8.00	7.17	− 6.32	< 0.001
Wait for what kind of exam	9.62	5.85	4.32	2.40	5.30	5.91	− 5.31	< 0.001
Whether to be hospitalized	9.85	5.67	4.12	2.37	5.73	5.72	− 5.75	< 0.001
Current status	10.78	9.03	5.52	3.68	5.27	8.80	− 4.28	< 0.001
Others	2.03	6.00	0.22	0.52	1.82	6.09	− 1.95	0.052

Table 4 shows a significant reduction in response time to answer questions (e.g., can I eat, wait for what kind of exam, and whether to be hospitalized) asked by patients and their families after setting up the ER digital bedside card (204.58 ± 139.92 vs. 8.28 ± 5.76, *P* < 0.001).

**Table 4:** Comparison of response time (seconds) to answer questions asked by patients and their families before and after setting up the ER digital bedside card.

	Pretest		Posttest		Pretest–posttest		Z	*P*
	M	SD	M	SD	M	SD		
Response time to answer questions	204.58	139.92	8.28	5.76	196.30	139.35	− 6.74	< 0.001

Table 5 shows a significant increase in ER staff satisfaction with patient information and patient identity and a decreased workload of ER staff after setting up the ER digital bedside card.

**Table 5:** Comparison of ER staff satisfaction before and after setting up the ER digital bedside card.

	Pretest		Posttest		Pretest–posttest		Z	*P*
	M	SD	M	SD	M	SD		
Patient information	2.98	1.02	4.60	0.56	− 1.617	1.166	− 6.23	< 0.001
Patient identity	3.05	1.02	4.67	0.48	− 1.617	1.209	− 5.97	< 0.001
Decreased workload of ER staff	2.63	1.02	4.40	0.67	− 1.767	1.184	− 6.25	< 0.001

## Discussion

The traditional ER bedside card (Fig. 1) used in the past was inconvenient, and patient information could not be immediately updated^2^. Therefore, we set up an ER digital bedside card that provides patient information, location, the anticipated procedure, admission status and health education information in real time.

After setting up the ER digital bedside card, our study showed that the number of round trips from the nursing station to the bedside was significantly reduced. This is a logical situation as the information provided by the ER digital bedside card can reduce the concerns of patients and their families. For example, if patients and their families want to know whether the patient can eat, they can see the ER digital bedside card. Instead of looking at the ER digital bedside card, they may ask the ER staff directly, and the ER staff can answer based on the ER digital bedside card. In addition, the staff do not need to return to the nursing station to query the computer or use memory to answer, which may lead to incorrect answers.

The total number of questions asked by patients and their family members was significantly reduced after setting up the ER digital bedside card. This makes sense because important frequently asked questions, such as whether the patient can eat, what reports the patient is waiting for, what exams are upcoming, whether the patient needs to be hospitalized, and the current status, can be answered. Although some of the questions are similar, according to the content of the ER digital bedside card, patients and their families can obtain answers, which can reduce the number of questions they have to ask ER colleagues and can reduce their anxiety and restlessness.

After setting up the ER digital bedside card, there was a significant reduction in response time to answer frequently asked questions (whether to eat, what kind of exam the patient was waiting for, and whether the patient needed to be hospitalized). In the past, when patients and family members wanted to know if the patient could eat, they might directly ask a passing doctor or nurse, which would interrupt their medical work. ER staff can answer them from memory, but there may be errors in their memory, or they may have to return to the nursing station to check the computer, which takes time. In general, if a passing doctor or nurse is not sure whether the patient can eat, they will write it down first, wait until the bedside work is over, return to the nursing station to inquire, and then return to the bedside and tell the patient and family members. It takes more than 10 min to reply to a patient's question in clinical practice. The design of this study assumes that we do not know whether the patient can eat. In this study, the ER staff were asked to put down their work and return to the nursing station to inquire, including opening the medical order page, entering the account password, finding the patient's medical record, determining whether they could eat, and then returning to the bedside to tell the patient and family members. The response time was approximately 200 s.

After setting up the ER digital bedside card, we found a significant increase in ER staff satisfaction with patient information and identity and a decreased workload of the ER staff. This is also very much in line with our expectations. To establish digital bedside cards, we must upgrade the electronic medical orders and create many important medical orders. For example, with regard to the question "Can I eat?", it is necessary to create a structured medical order (e.g., nothing by mouth, nothing by mouth except medication, light diet and normal diet) in the HIS to automatically bring it to the ER digital bedside card. Discussions with the information department, nurses, escorts, and doctors and reaching a consensus with all staff takes time. However, after finishing, there is no need for emergency physicians to do extra work, and it is convenient for statistics. Likewise, do not resuscitate (DNR) orders and most exams are already structured medical orders, and the HIS automatically brings them to the ER digital bedside card with no further work needed.

Our study may be the first article about setting up digital bedside cards in the ER, with the exception of our previous work. Patients on the ward have received medical management, and their condition is less urgent than patients in an emergency room. Therefore, the design function of the ward digital bedside card may be different from that of the ER digital bedside card. Our findings demonstrate that the provision of ER digital bedside cards can reduce the labour of ER colleagues, including the number of round trips to and from the bedside, questions asked by patients and their family members, and the response time to answer frequently asked questions by patients and their family members and can increase the satisfaction of ER colleagues. It can also help other ERs set up ER digital bedside cards in the future to reduce the burden of all ER colleagues and improve the quality of emergency medical care, thereby becoming an important milestone in the development of smart ERs in the future. We plan to implement an early warning system in the ER that can also be displayed on digital bedside cards. The digital bedside card will turn red if the warning is critical^7^.

There are several limitations of this study. First, the workload of emergency room staff is very heavy, and it is difficult to calculate the numbers accurately. We encourage colleagues to calculate as correctly as possible but to avoid increasing their workload. We explained this to the participants in great detail, and if they had any questions about the questionnaire, they could call for help. Second, even though it is a simple idea, the ER digital bedside card still takes considerable money and time to implement in practice. It includes hardware equipment, software settings, digital bedside card content displays, and structured medical orders and meets the needs of medical staff and patients' families. However, it cannot receive medical insurance reimbursement. Third, this study did not assess patient and family satisfaction due to difficulty explaining and collecting pretest/posttest questionnaires. Fourth, although this was a prospective study, it was performed in only one medical centre ER in Taiwan. The policies and conditions of ERs vary from country to country, and the card may not be practical in other countries. However, from the research results, we believe that the ER digital bedside card could greatly improve the efficiency of the emergency department. Therefore, large-scale research should be planned to confirm these benefits.

## Conclusions

The ER digital bedside card, which provides real-time patient information, is a simple but important idea. It took 2 years of hard work to set it up in our ER. We are pleased to demonstrate that the ER digital bedside card can reduce the workload of ER staff, provide patients and their families with useful information, and greatly improve ER staff satisfaction. It is an important milestone in the future development of a smart ER.

## Supplementary Information

Supplementary Information.

## Data Availability

The datasets used and/or analysed during the current study available from the corresponding author on reasonable request.

## References

[1] Ku NW (2021). Bed-to-bed transfer program among patients who need hospitalization in a crowded emergency department in Taiwan. Int. J. Health Policy Manag..

[2] Chang WH, Huang MK, Lin JP, Chien DK (2019). A real-time digital bedside card of emergency room: A new concept. Health Technol..

[3] Wang Z, Wang Y, Huang Z, Wang J (2021). Design and implementation of multifunctional interactive electronic bedside card system for inpatients based on internet of things technology. Zhongguo Yi Liao Qi Xie Za Zhi.

[4] Wang Z, Wang Y, Huang Z, Wang J (2021). Design and implementation of multifunctional interactive electronic bedside card system for inpatients based on internet of things technology. Zhongguo Yi Liao Qi Xie Za Zhi.

[5] Lin, C. Y., Ma, T. Y., Lin, C. L., Hsu, M. C. & Hou, T. W. An electronic hospital bedside-card prototype using RFID and DVB-MHP platform in *2009 10th International Symposium on Pervasive Systems, Algorithms, and Networks* 390–393 (IEEE, 2009).

[6] Kwak SG, Kim JH (2017). Central limit theorem: the cornerstone of modern statistics. Korean J. Anesthesiol..

[7] Usman OA, Usman AA, Ward MA (2019). Comparison of SIRS, qSOFA, and NEWS for the early identification of sepsis in the emergency department. Am. J. Emerg. Med..

