# Supplementary material for: A pilot study of digital bedside cards in the emergency room of a medical centre in Taiwan
Source: Sci Rep. 2023 Apr 28;13:6965. doi: 10.1038/s41598-023-34234-4 (PMC10147724; doi:10.1038/s41598-023-34234-4)
Supplement: Supplementary file 1 — Supplementary Information. [file 41598_2023_34234_MOESM1_ESM.docx]

**An ER digital bedside card efficacy and satisfaction survey (pretest)**

Hi. We are planning to build a digital bedside card in the emergency room (ER). To study whether this plan can achieve our expected goals, we need your professional and rich work experience to assist us in a questionnaire survey. Thank you very much for your assistance and cooperation despite your busy schedule! The questions in this questionnaire should be answered based on your actual daily work situation. It is recommended that you read the questions first and then pay attention to the work situation on the next working day to facilitate completion. Thank you!

Date： Signature: __________________

| 1. Job title  ⬜ Physician ⬜ Nurse ⬜ Nurse practitioner ⬜ Escort  2. Seniority  ⬜ 0-5 years ⬜ 6-10 years ⬜ 11-15 years ⬜ ≧16 years  3. Work shift：  ⬜ 07:00-19:00 ⬜ 19:00-07:00  ⬜ 08:00-16:00 ⬜ 16:00-24:00  ⬜ 24:00-08:00  4. Work area：（Multiple choice）  ⬜ Internal medicine clinic ⬜ Surgical clinic  ⬜ First observation unit ⬜ Second observation unit  ⬜ Third observation unit  5. Number of cases involved in this shift  ⬜ under 20 ⬜ 20 to 40 ⬜ above 40 (patients)  6. Used to identify patient by（multiple choice）  ⬜ Ask ⬜ Patient identification wristband  ⬜ **Bed card** (under the bed) ⬜ Bedside card（on the wall, as in picture 1) | 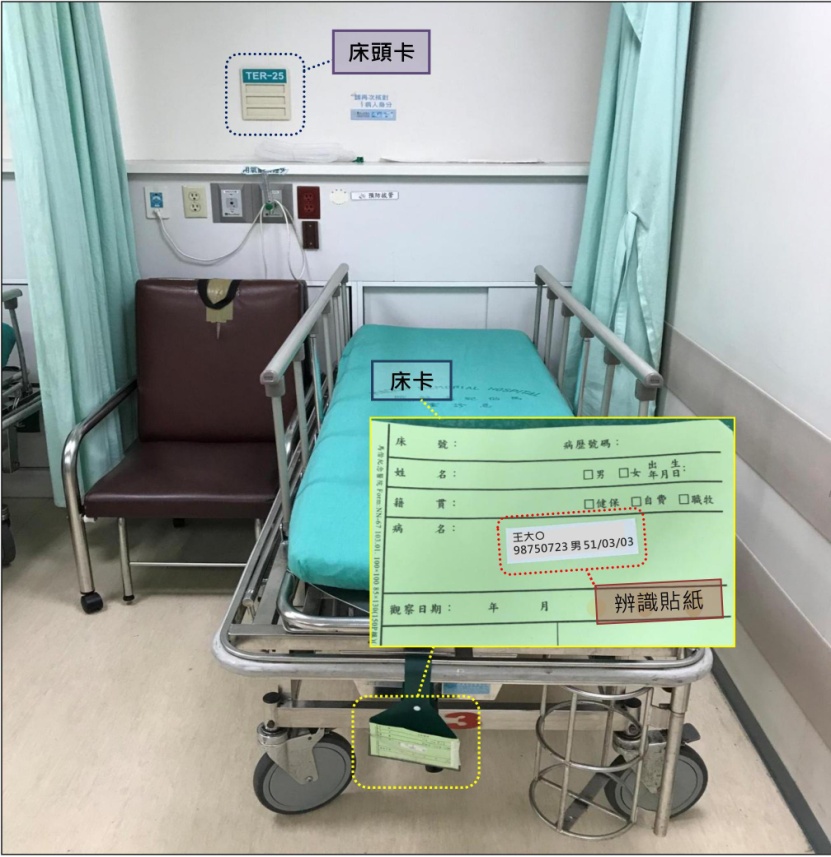 |
| --- | --- |
|  | Picture 1：Traditional Bedside Card |

7. Have you ever found a mismatch between a patient's real bed (position) and the computer system?

⬜ No

⬜ YES, because the last patient at that location was not closed on the computer system, but he or she left the emergency department; the frequency was approximately ____ times/day or______ times/month (choose one).

⬜ YES, due to a delay in updating the location of patient in the computer system; the frequency was approximately ____ times/day or______ times/month（choose one）

⬜ YES，due to patient misalignment; the frequency was approximately ____ times/day or______ times/month（choose one）

⬜ YES, other reason such as _________________; the frequency was approximately ____ times/day or______ times/month（choose one）

8. Reason for round trip from nursing station to the bedside: (multiple choice)

⬜ Treatment ⬜ Answer questions from patient/family

⬜ Explain current status ⬜ Explain result of examination ⬜ Other__________________

9. In today’s shift, how many round trips did you make from the nursing station to the bedside? _______ times

10. In today’s shift, how many questions were received from patients/families? ________times

11. Based on question 10, please estimate the number of questions asked by patients/family members.

(Can I eat, ______times；Wait for what, ______times；Wait for what kind of exam, ______times；Whether to be hospitalized, ______times；Current status, ______times；Other, ______times)

12. Please estimate the response time to answer questions asked by patients/families, including the time spent on round trips between bedsides and stations and checking the computer system.

＿＿＿＿＿(mins)＿＿＿＿(sec)

13. Satisfaction with traditional bedside cards

| Information provided | ⬜ Very Satisfied ⬜ Satisfied ⬜ Normal ⬜ Dissatisfied ⬜ Very Dissatisfied |
| --- | --- |
| Identify patient | ⬜ Very Satisfied ⬜ Satisfied ⬜ Normal ⬜ Dissatisfied ⬜ Very Dissatisfied |
| Round trip from nursing station to the bedside | ⬜ Very Satisfied ⬜ Satisfied ⬜ Normal ⬜ Dissatisfied ⬜ Very Dissatisfied |

| 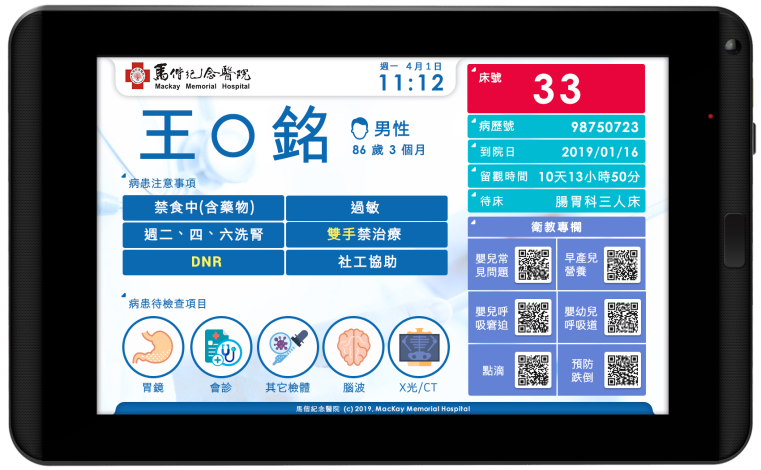 | 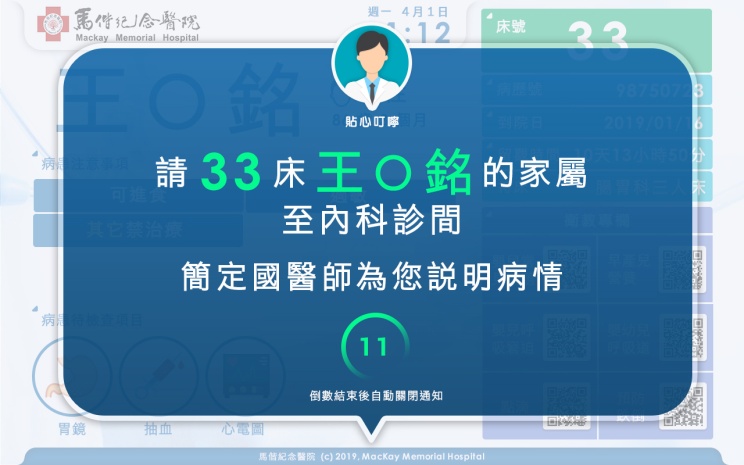 |
| --- | --- |

Picture 2: ER digital bedside card

14. Picture 2 is the schematic diagram of the ER digital bedside card. Please select the expectation.

| The digital bedside card can help guide patients to the right bed | ⬜ Very Satisfied ⬜ Satisfied ⬜ Normal ⬜ Dissatisfied ⬜ Very Dissatisfied |
| --- | --- |
| The digital bedside card provides information importantly and clearly | ⬜ Very Satisfied ⬜ Satisfied ⬜ Normal ⬜ Dissatisfied ⬜ Very Dissatisfied |
| Digital bedside cards can decrease round trips from the station to the bedside | ⬜ Very Satisfied ⬜ Satisfied ⬜ Normal ⬜ Dissatisfied ⬜ Very Dissatisfied |
| Compared to traditional bedside cards, digital bedside cards help identify patients more correctly | ⬜ Very Satisfied ⬜ Satisfied ⬜ Normal ⬜ Dissatisfied ⬜ Very Dissatisfied |
| The digital bedside card can improve the efficiency of work | ⬜ Very Satisfied ⬜ Satisfied ⬜ Normal ⬜ Dissatisfied ⬜ Very Dissatisfied |
| In general, the digital bedside card can improve the ER | ⬜ Very Satisfied ⬜ Satisfied ⬜ Normal ⬜ Dissatisfied ⬜ Very Dissatisfied |

15. Following question 12, please estimate the response time to answer questions asked by patients/family members **if** digital bedside cards are applied

＿＿＿＿＿(mins)＿＿＿＿(sec)

**An ER digital bedside card efficacy and satisfaction survey (posttest)**

Hi. We are planning to build a digital bedside card in the emergency room (ER). To study whether this plan can achieve our expected goals, we need your professional and rich work experience to assist us in the questionnaire survey. Thank you very much for your assistance and cooperation despite your busy schedule! Some questions in this questionnaire need to be answered based on your actual daily work situation. It is recommended that you read the questions first and then pay attention to the work situation on the next working day to facilitate completion. Thank you!

Date： Signature: __________________

| 1. Job title  ⬜ Physician ⬜ Nurse ⬜ Nurse practitioner ⬜ Escort  2. Seniority  ⬜ 0-5 years ⬜ 6-10 years ⬜ 11-15 years ⬜ ≧ 16 years  3. Work shift：  ⬜ 07:00-19:00 ⬜ 19:00-07:00  ⬜ 08:00-16:00 ⬜ 16:00-24:00  ⬜ 24:00-08:00  4. Work area：（multiple choice）  ⬜ Internal medicine clinic ⬜ Surgical clinic  ⬜ First observation Unit ⬜ Second observation unit  ⬜ Third observation unit  5. Number of cases involved in this shift  ⬜ under 20 ⬜ 20 to 40 ⬜ above 40 (patients)  6. Used to identify patient by（multiple choice）  ⬜ Ask ⬜ Patient identification wristband  ⬜ Bed card ⬜ **Digital bedside card** (picture 1) | 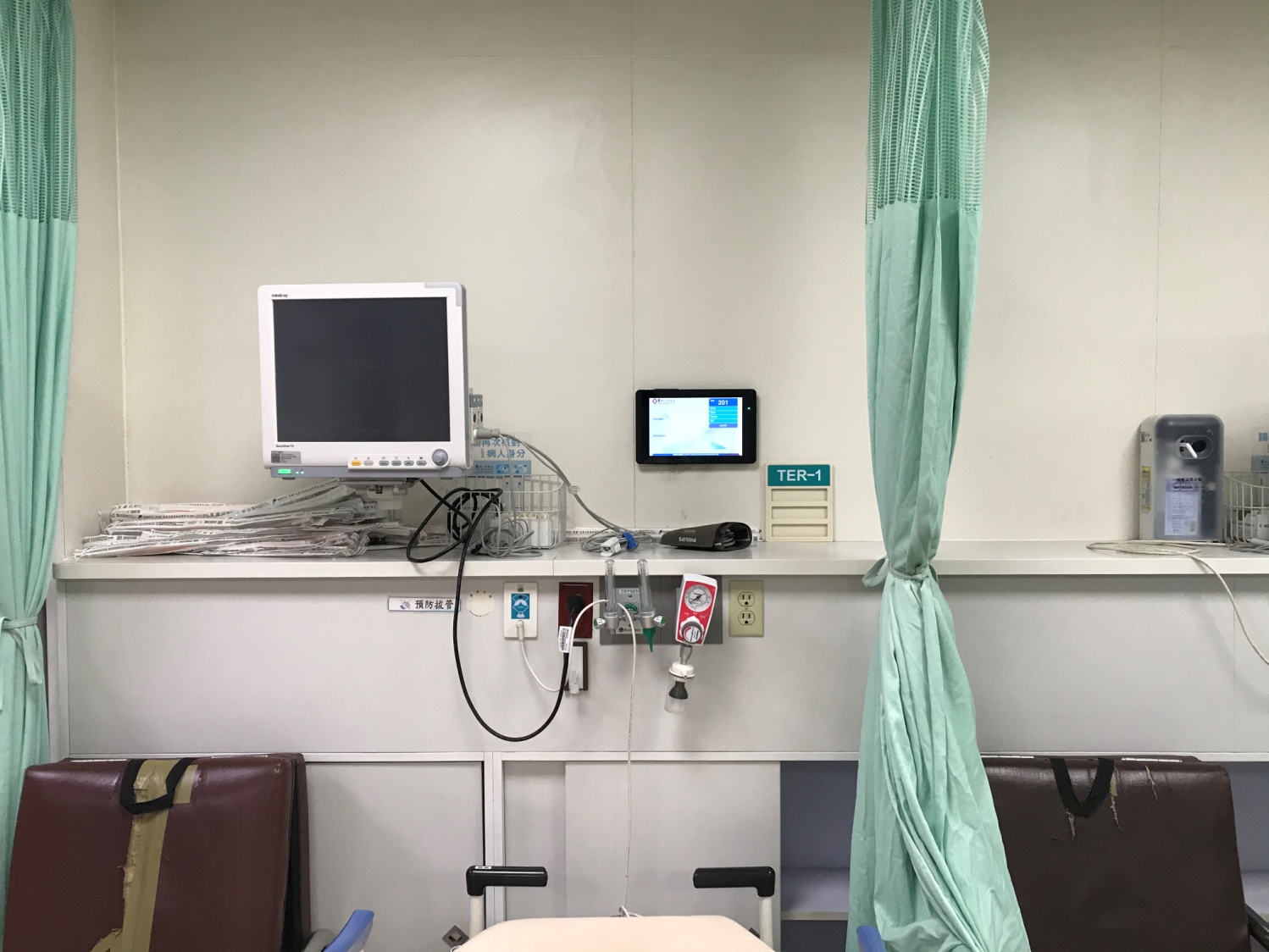 |
| --- | --- |
|  | Picture 1：Digital Bedside Card |

7. Have you ever found a mismatch between a patient's real bed (position) and the computer system?

⬜ No

⬜ YES, because the last patient at that location was not closed on the computer system, but he or she left emergency department; the frequency was approximately ____ times/day or______ times/month（choose one）

⬜ YES, due to a delay in updating the location of patient on computer system; the frequency was approximately ____ times/day or______ times/month（choose one）

⬜ YES, due to patient misalignment; the frequency was approximately ____ times/day or______ times/month (choose one)

⬜ YES, other reason such as __________________; the frequency was approximately ____ times/day or______ times/month（choose one）

8. Reason for round trip from nursing station to the bedside: (multiple choice)

⬜ Treatment ⬜ Answer questions from patient/family

⬜ Explain current status ⬜ Explain result of examination ⬜ Other__________________

9. In today’s shift, how many round trips did you make from the nursing station to the bedside? _______ times 10. In today’s shift, how many questions were received from patients/families? ________times 11. Based on question 10, please estimate the number of questions asked by patients/family members

(Can I eat, ______times；Wait for what, ______times；Wait for what kind of exam, ______times；Whether to be hospitalized, ______times；Current status, ______times；Other, ______times)

12. Please estimate the response time to answer questions asked by patients/families, including the time spent on round trips between the bedside and the station and checking the computer system.

＿＿＿＿＿(mins)＿＿＿＿(sec)

13. Satisfaction with traditional bedside cards

| Information provided | ⬜ Very Satisfied ⬜ Satisfied ⬜ Normal ⬜ Dissatisfied ⬜ Very Dissatisfied |
| --- | --- |
| Identify patient | ⬜ Very Satisfied ⬜ Satisfied ⬜ Normal ⬜ Dissatisfied ⬜ Very Dissatisfied |
| Round trip from nursing station to the bedside | ⬜ Very Satisfied ⬜ Satisfied ⬜ Normal ⬜ Dissatisfied ⬜ Very Dissatisfied |

| 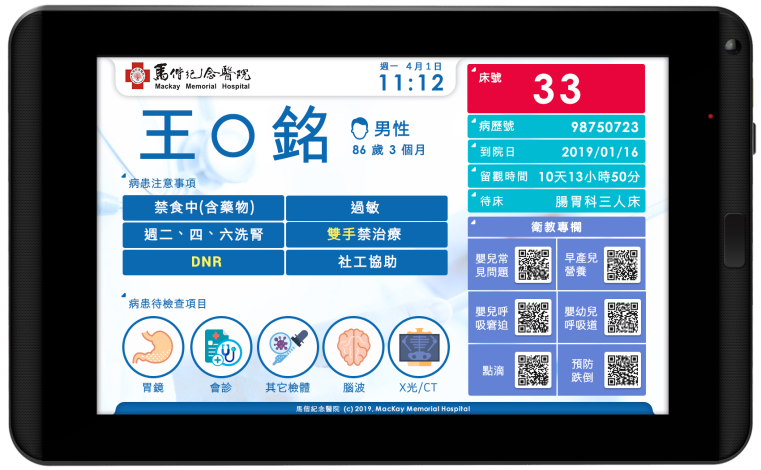 | 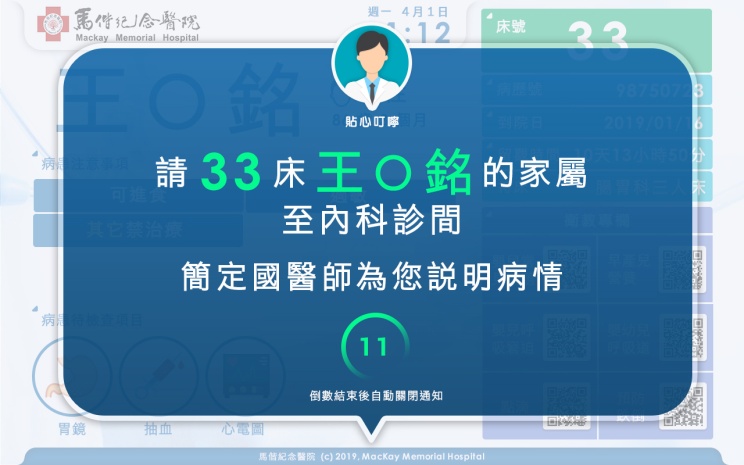 |
| --- | --- |

Picture 2: ER digital bedside card

14.Picture 2 is the schematic diagram of the ER digital bedside card. Please select the expectation.

| The digital bedside card can help guide patients to the right bed | ⬜ Very Satisfied ⬜ Satisfied ⬜ Normal ⬜ Dissatisfied ⬜ Very Dissatisfied |
| --- | --- |
| Digital bedside cards provide information importantly and clearly | ⬜ Very Satisfied ⬜ Satisfied ⬜ Normal ⬜ Dissatisfied ⬜ Very Dissatisfied |
| Digital bedside cards can decrease round trips from the station to bedside | ⬜ Very Satisfied ⬜ Satisfied ⬜ Normal ⬜ Dissatisfied ⬜ Very Dissatisfied |
| Compared to traditional bedside cards, digital bedside cards help identify patients more correctly | ⬜ Very Satisfied ⬜ Satisfied ⬜ Normal ⬜ Dissatisfied ⬜ Very Dissatisfied |
| Digital bedside cards can improve the efficiency of work | ⬜ Very Satisfied ⬜ Satisfied ⬜ Normal ⬜ Dissatisfied ⬜ Very Dissatisfied |
| In general, digital bedside cards can improve the ER | ⬜ Very Satisfied ⬜ Satisfied ⬜ Normal ⬜ Dissatisfied ⬜ Very Dissatisfied |

15. Following question 12, please estimate the response time to answer questions asked by patients/families **after** digital bedside cards are applied

＿＿＿＿＿(mins)＿＿＿＿(sec)
